# Pharmacodynamic and Therapeutic Investigation of Focused Ultrasound-Induced Blood-Brain Barrier Opening for Enhanced Temozolomide Delivery in Glioma Treatment

**DOI:** 10.1371/journal.pone.0114311

**Published:** 2014-12-09

**Authors:** Hao-Li Liu, Chiung-Yin Huang, Ju-Yu Chen, Hay-Yan Jack Wang, Pin-Yuan Chen, Kuo-Chen Wei

**Affiliations:** 1 Department of Electrical Engineering, Chang-Gung University, Taoyuan, Taiwan; 2 Department of Neurosurgery, Chang-Gung Memorial Hospital, Linkou, Taiwan; 3 Department of Biological Sciences, National Sun Yat-sen University, Kaohsiung, Taiwan; 4 Healthy Aging Research Center, Chang-Gung University, Taoyuan, Taiwan; The Ohio State University, United States of America

## Abstract

Focused ultrasound (FUS) exposure with the presence of microbubbles has been shown to transiently open the blood-brain barrier (BBB), and thus has potential to enhance the delivery of various kinds of therapeutic agents into brain tumors. The purpose of this study was to assess the preclinical therapeutic efficacy of FUS-BBB opening for enhanced temozolomide (TMZ) delivery in glioma treatment. FUS exposure with microbubbles was delivered to open the BBB of nude mice that were either normal or implanted with U87 human glioma cells. Different TMZ dose regimens were tested, ranging from 2.5 to 25 mg/kg. Plasma and brain samples were obtained at different time-points ranging from 0.5 to 4 hours, and the TMZ concentration within samples was quantitated via a developed LC-MS/MS procedure. Tumor progression was followed with T2-MRI, and animal survival and brain tissue histology were conducted. Results demonstrated that FUS-BBB opening caused the local TMZ accumulation in the brain to increase from 6.98 to 19 ng/mg. TMZ degradation time in the tumor core was found to increase from 1.02 to 1.56 hours. Improved tumor progression and animal survival were found at different TMZ doses (up to 15% and 30%, respectively). In conclusion, this study provides preclinical evidence that FUS-BBB opening increases the local concentration of TMZ to improve the control of tumor progression and animal survival, suggesting the potential for clinical application to improve current brain tumor treatment.

## Introduction

At least 23,000 patients are diagnosed with malignant primary brain or other CNS cancers in the United States each year, and nearly half of the patients develop high-grade glioma or glioblastoma multiforme (GBM) [1]. GBM has high mortality and typically results in death in the first several months after diagnosis. GBM patients first undergo debulking surgery to remove most of the tumor mass, followed by chemotherapy and/or radiation therapy. A phase-III randomized trial found that the prognosis of GBM patients remains poor after debulking surgery and radiation, with a median survival time of only 12 months [2].

Chemotherapy is considered to be an important treatment modality for malignant brain tumors [3], and currently the most important chemotherapy agent administered to control glioma progression is temozolomide (TMZ). TMZ is an alkylating agent of the imidazotetrazine series that possesses strong antineoplastic activity against high-grade glioma [4], [5]. It has been reported that TMZ exerts its antitumor activity by being irreversibly converted to the linear triazine 5-(3-methyltriazen-1-yl) imidazole-4-carboxamide (MTIC). MTIC is believed to be the major antitumor effector due to its potent alkylating activity [6]. However, to date, the improvement and overall success of TMZ administration remains limited and far from satisfactory in comparison with the treatment and management of other tumor types. Clinical trials have shown that the median survival in patients treated with radiation plus TMZ was limited to 3 to 4 months longer than that in patients treated by radiation alone, which is far from satisfactory [7], [8].

It is believed that one major obstacle to effective treatment is the high vascularity and heterogeneous permeability of brain tumors. Contrast-enhanced areas only partially represent the tumor-cell distribution [9] and autopsy studies have demonstrated glioblastoma cells at great distances from the enhancing regions of tumors [10], [11]. It is already well-known that tumor-associated BBB breakdown is highly heterogeneous, with the tumor core often being the most permeable compared to the impermeable proliferating tumor periphery [12]–[15]. In addition, a major dilemma is that by systematically increasing the chemotherapeutic agent concentration in an attempt to increase the receiving dose to entire tumor regions, substantial systemic toxicity of chemotherapy negatively impacts the already poor quality-of-life during the patient's remaining life span [16]. One potential strategy is therefore to combine local or targeted drug delivery techniques to enhance local drug concentration with a systemic dose of chemotherapeutic agent within the limits tolerated by the body.

Focused ultrasound (FUS) exposure combined with microbubbles has been shown to transiently open the blood-brain barrier (BBB) at the targeted brain region, thus offering a new opportunity for local drug delivery to brain tumors [9], [17]–[20]. Intravenous administration of microbubbles significantly reduces the ultrasound exposure level required to prevent brain tissue damage, and specifically permeates the targeted CNS capillary since the triggered MB-ultrasound interaction is mainly confined to within the blood vessel [20]. Compared to alternative approaches such as modified lipophilic chemicals or carotid infusion of hypertonic solution [15], [21], FUS thus presents a competitive and attractive alternative for local induction of BBB disruption to increase the local concentrations of chemotherapeutic agents in GBM. Previously, we reported that FUS-BBB opening remarkably increases the concentration of BCNU (2–3 fold), a clinically approved GBM treatment, thereby improving the therapeutic efficacy in glioma-bearing rats [22]. Also, we recently reported that FUS-BBB opening combined with temozolomide administration can provide successful tumor progression control and survival improvement in glioma-bearing rats [23]. Yet, because TMZ has a relatively fast pharmacodynamic rate and is quickly metabolized (half-life of approximately <1.5 hours in a small animal [24]), the previous study did not measure TMZ in brain tissues and plasma, and it is still unknown how FUS-BBB opening affects the local deposition and dynamic concentration change of TMZ in the targeted tissue or plasma.

The purpose of this study was to investigate the pharmacodynamic change and therapeutic efficacy of TMZ when administered with FUS-BBB opening. We hypothesized that the temporary disruption of the tight junctions in brain capillaries would promote local TMZ permeability and deposition in the targeted brain site. Human glioma cell-bearing mice were used as the animal model to better mimic clinical GBM behavior. Techniques to measure TMZ concentration in brain tissues and plasma have been developed, and assessment of TMZ pharmacodynamic change and quantification was performed to clarify the effect of FUS-BBB opening of the efficacy of glioma treatment. We present evidence that FUS-BBB opening can be beneficial for increasing the local deposition of chemotherapeutic agent, thus improving therapeutic efficacy, including tumor shrinkage and animal survival.

## Materials and Methods

### U87 glioma animal model

U87 mice glioma cells were cultured at 37°C in a humidified 5% CO_2_ atmosphere in minimum essential median (MEM) supplemented with 10% fetal bovine serum and 1% penicillin/streptomycin (Invitrogen). Cells were harvested by trypsinization, washed once with phosphate-buffered saline (PBS), and resuspended (1×10^5^ cell/µl) in MEM for implantation into the striatum of mouse brains. Pathogen-free male NU/NU mice (5 to 7 weeks old) were purchased from BioLASCO (Taiwan). Mice were housed and maintained in a controlled environment and all procedures were performed in accordance with the experimental animal care guidelines of the Animal Committee of Chang Gung University. To implant U87 tumor cells, we anesthetized animals with 2% isoflurane gas and immobilized them on a stereotactic frame. A sagittal incision was made through the skin overlying the calvarium, and a 23G needle was used to create a hole in the exposed cranium 1.5 mm anterior and 2 mm lateral to the bregma. Three microliters of U87 glioma cell suspension were injected at a depth of 2 mm from the brain surface. The injection was performed over a 3-minute period, and the needle was withdrawn over another 2 minutes. The growth of the mouse brains was monitored by MRI for 10 days post tumor cell implantation.

### Focused Ultrasound Treatment

**Figure 1:**
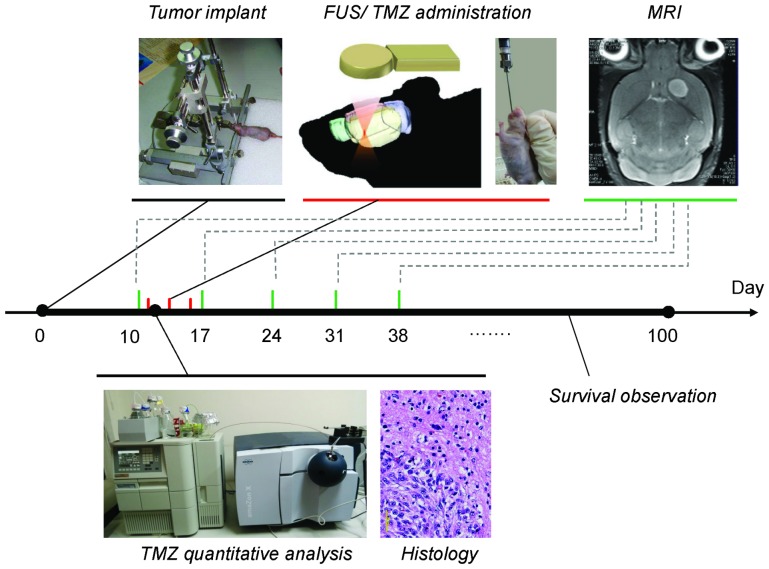
Conceptual diagrams and time course for experimental approach using focused ultrasound (FUS)-induced blood-brain barrier opening to enhance temozolomide (TMZ) delivery in a glioma animal model.

Fig. 1 illustrates the entire study design. A FUS transducer (Sonic Concepts Inc., Washington, USA; diameter  = 60 mm, radius of curvature  = 52 mm, frequency  = 500 kHz) was applied to generate concentrated ultrasound energy. An arbitrary function generator (33120A, Agilent, Palo Alto, CA) was used to produce the driving signal, which was fed to a radio frequency power amplifier (No. 500-009, Advanced Surgical Systems, Tucson, AZ) operating in burst mode. Animals were anesthetized with 2% isoflurane and immobilized on a stereotactic frame. The top of the cranium was shaved with clippers, and a PE-10 catheter was inserted into the tail vein. The animal was placed directly under an acrylic water tank (with a window of 4×4 cm^2^ at its bottom sealed with a thin film to allow entry of the ultrasound energy) with its head attached tightly to the thin-film window. SonoVue SF6-coated ultrasound microbubbles (2–5 µm, 4 µl/mouse; Bracco, Milan, Italy) were administered intravenously before treatment. The tumor-implant hemisphere brain site was then exposed to burst-tone mode ultrasound to locally open the BBB (electrical power  = 2–5 W; peak negative pressure  = 0.3–0.7 MPa; burst length  = 10 ms; pulse repetition frequency  = 1 Hz; exposure time  = 60 s).

### Quantitation of TMZ

The blood level of TMZ in mice was monitored using methods for analysis of plasma TMZ reported by Baker et al. [6] and Portnow et al. [25]. Animals were fed with TMZ (50 mg/kg), and euthanized after a designated time period. Once the mouse lost its tail pinch reflex, blood was aspired into a heparinized syringe by transcardiac puncture. The blood was immediately mixed with 5 volumes of 0.01N HCl. Twenty µL of acidified blood was mixed with 10 µL of IS (10 ng/µL) in a new sample vial in the presence of 100 µL of 0.01N HCl and vortexed, then centrifuged at 14,000 rpm for 10 min under 4°C. The supernatant was collected and extracted with 400 µL ethyl acetate twice. The drug-containing ethyl acetate was pooled in another new sample vial and dried under a gentle stream of nitrogen. Thereafter, the pellet was reconstituted in mobile phase A (see below) for LC-MS/MS analysis. The preparation of calibration standard for blood TMZ measurement was identical to the above method for drug-containing blood. The drug-free mouse blood was used and a suitable amount of TMZ and identical level of IS were both spiked and vortexed. The subsequent handlings were identical to the above process. The calibration range was prepared between 0.5 and 150 ng/µL.

TMZ in mice plasma/brain tissue was analyzed after euthanizing mice by injection of an overdose of equithesin. Plasma/tissue was aspirated through the Foramen Magnum into a syringe containing 6% HOAc and transferred to a vial prechilled on ice and containing additional HOAc, such that the final volume ratio of 6% HOAc∶CSF was 1∶5. The mixture was briefly vortexed and centrifuged at 14,000 rpm for 5 minutes at 4°C. Twelve microliters of supernatant was mixed with 10 µL IS (500 ng/mL in MeOH/0.5% HOAc (50∶50)) and 100 µL of 0.5% HOAc in the HPLC sample vial for subsequent LC-MS/MS analysis. Standard concentrations for the analysis of TMZ in plasma/tissue were prepared over the range of 0.5–150 ng/ml in 0.5% HOAc.

The LC-MS/MS system consisted of a Waters 2695 separation module for HPLC with an outflow that was coupled to the electrospray ionization source of an amaZon X ion trap mass spectrometer (Bruker Daltonics). TMZ was eluted from an Ascentis Express C18 column (2.1×50 mm; particle size 2.7 µm) with an isocratic mobile phase (14% acetonitrile and 0.1% formic acid) at a flow rate of 0.2 mL/min. The temperature of the column was maintained at 30°C, whereas the temperature of the autosampler was kept at 5°C. The mass spectrometer was operated in positive ion mode. TMZ and IS were detected by multiple-reaction-monitoring (MRM). The transition from precursor to product ion for TMZ occurs from m/z 194.9 to m/z 137.8, and from m/z 197.9 to m/z 139.9 for IS. Each chromatography run took approximately 10 minutes. A 20-µL aliquot was injected into the column for analysis of TMZ in both plasma and brian tissue. Quantitative analysis of TMZ was carried out with QuantAnalysis (Bruker Daltonics).

### Animal Experiment Design

All animal experiments were approved by the Animal Committee of Chang Gung University and adhered to the experimental animal care guidelines. A total of 294 mice were used, including normal (n = 50) and tumor-bearing mice (n = 244). Experiments were divided into three groups. In experimental group 1, the primary aim was to assess if FUS-BBB opening promoted TMZ penetration and deposition in normal mice (n = 27), while a small number of normal animals (n = 15) were employed to test and determine the appropriate FUS power range (powers of 2 and 5 W were employed and evaluated). The majority of normal animals were divided into two groups, without FUS-BBB opening (n = 6) and with FUS-BBB opening (n = 6), and the brain samples with the TMZ concentration were analyzed and quantified using LC-MS/MS analysis (active pharmaceutical ingredients of temozolomide obtained from Lotus Pharmaceutical Co. Ltd, TAIWAN, a generic drug of Temozolomide, Schering-Plough, NJ, USA). All animals were sacrificed and the brain samples were preserved 2 hour after TMZ administration.

In experimental group 2, the aim was to dynamically quantitate the TMZ concentration in tumor-bearing animals to verify whether FUS-BBB opening affects TMZ depositions and pharmacodynamics in the brain. Animals were divided into two groups: (1) TMZ administration only (n = 39) and (2) TMZ administration following FUS-BBB opening (n = 43). Treatment session was started when the implanted tumor mass had grown to be MRI-detectable and the experiments were started. Animals were given 50 mg/kg of TMZ (day 1 after the 1^st^ MRI screening) and in the combined TMZ with BBB-opening group, FUS exposure was performed prior to oral delivery of TMZ on the same day. Animals were immediately sacrificed, and brain samples (including tumor and contralateral brain tissues) as well as plasma (total of 500 µL per each animal) were obtained for TMZ quantification. (Only 1 sample per each mouse tissue or blood)

In experimental group 3, the aim was to evaluate the therapeutic efficacy of TMZ combined with FUS exposure in tumor-bearing mice. TMZ uptake was performed 10 days after U87 glioma cell implantation. TMZ was orally administered three times (days 1, 3, and 5 after the 1^st^ MRI screening), and the tumor progression and survival were both longitudinally followed. For animal groups with BBB-opening, FUS exposure was conducted twice (day 1 and 5 after the 1^st^ MRI screening; before oral administration of TMZ). Animals were divided into 7 sub-groups: (1) sham (without TMZ administration)(n = 27); (2) TMZ  = 2.5 mg/kg per day for 3 days (n = 18); (3) TMZ  = 5 mg/kg per day for 3 days (n = 13); (4) TMZ  = 25 mg/kg per day for 3 days (n = 8); (5) FUS-BBB opening only, without TMZ administration (n = 16); (6) TMZ  = 2.5 mg/kg per day for 3 days, plus FUS-BBB opening (n = 17); and (7) TMZ  = 25 mg/kg per day for 3 days, plus FUS-BBB opening (n = 8). The detailed experimental timeline is shown in Fig. 1. In tumor-bearing animal groups, humane endpoint was set to the MRI-measured tumor volume greater than 200 mm^3^ (∼40% of total brain volume; MRI was acquired once per week) or higher than 20% body weight drop during one week (animals were weigh twice per week), and animals meet humane endpoint were euthanized by carbon dioxide inhalation.

### Magnetic Resonance Imaging and Analysis

Tumor-bearing mice were followed to monitor the progression of brain tumors. All MRI images were acquired on a 7-Tesla magnetic resonance scanner (Bruker ClinScan, Germany) and a 4-channel surface coil was used on the top of the mouse brain. The animals were anesthetized through inhalation of 2% isoflorane throughout the MRI process, placed in an acrylic holder and positioned in the center of the magnet. In the tumor animal experiment group, tumor size was quantified using turbo-spin-echo based T2-weighted images with the following parameters: pulse repetition time (TR)/echo time (TE)  = 2000/41 ms; FOV  = 33×50 mm^2^ (162×320 pixels); slice thickness  = 0.5 mm. The relative tumor size was estimated by measuring the single image slide containing the maximum tumor area, and animals were longitudinally imaged every 7 days for up to 38 days after the 1^st^ MRI screening.

### Pharmacodynamic analysis

The dynamic concentration change of TMZ in plasma, CSF, and brain tissues was analyzed by non-compartmental methods [26]. Samples in group 2 animals were collected at four time-points: 0.5, 1, 2, and 4 hrs. The peak concentrations along the time profile were obtained, and the time of the peak concentration was also denoted. The TMZ degradation constant *K* was calculated as the negative of the slope of the log-linear terminal portion of the plasma concentration-time curve using linear regression (PRISM, GraphPad Inc., CA, USA). The time required for 50% degradation of TMZ from peak concentration was estimated by ln(2)/*K* [26].

### Histological examination

To confirm and demonstrate the FUS-BBB opened region in brain tissues, Evans Blue (EB) dye (3% in saline) was injected intravenously (4 µL/mouse) and selected animals were sacrificed two hours later. Tissues were prepared for histology after *in vivo* MRI analysis. Histopathology was performed on 10-µm sections from paraformaldehyde-fixed, paraffin-embedded brains. In the parametric testing group of normal animal experiments, EB dye was administered after MRI and before animal sacrifice for gross observation of the BBB disrupted region. Animals were sacrificed four hours after dye injection.

Microscopy was performed using a CCD digital camera mounted on a microscope (TissueFAX Plus, TissueGnostics, Austria). Hematoxylin and eosin (H&E) staining was conducted to evaluate the ultrasound-induced brain tissue damage. For brain-tumor implant animals, H&E staining was also carried out to histologically confirm the tumor progression.

### Statistical analysis

The statistical significance of increased signal intensity was determined using a two-tailed unpaired *t* test, with *p*<0.05 considered to be significant. In experimental group 3, the Kaplan-Meier method was used to perform animal survival analysis. Statistical significance was calculated using the Mantel-Cox test, with statistical significance assumed at p<0.05. The different treatment groups were compared in terms of median survival time (in days), increase in median survival time (IST_median_; in %), mean survival time (in days), and increase in mean survival time (IST_mean_; in %).

## Results

**Figure 2:**
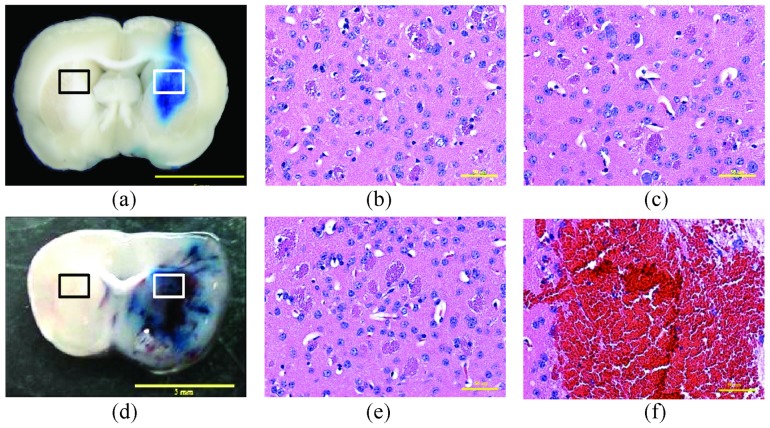
(a, d) Representative Evans Blue (EB) dye staining in animal brains after FUS-BBB opening, and HE stains at 2 W (upper) and 5 W (lower), respectively. (b, e) HE staining of contralateral brain regions. (c, f) HE staining of BBB-opened regions. Bar  = 50 µm.

We first evaluated the influence of FUS exposure power level on BBB opening. Fig. 2 demonstrates typical brain sections and HE stains after FUS-BBB opening with an exposure level of 2 or 5 W. An exposure power level of 2 W induced a successful BBB opening effect, confirmed by EB dye staining in the exposed brain hemisphere. HE stains also confirmed that the CNS cells did not show any pathological changes and were intact. When a higher exposure level of 5 W was applied, the BBB-opened regions spread toward a wider area, with RBCs extravasated in the exposure regions (both confirmed from gross sections and HE stains). When considering both successful BBB-opening and safety with minimal possible tissue hazard induced by FUS exposure, a FUS exposure level of 2 W was selected and applied in subsequent animal experiments.

**Figure 3:**
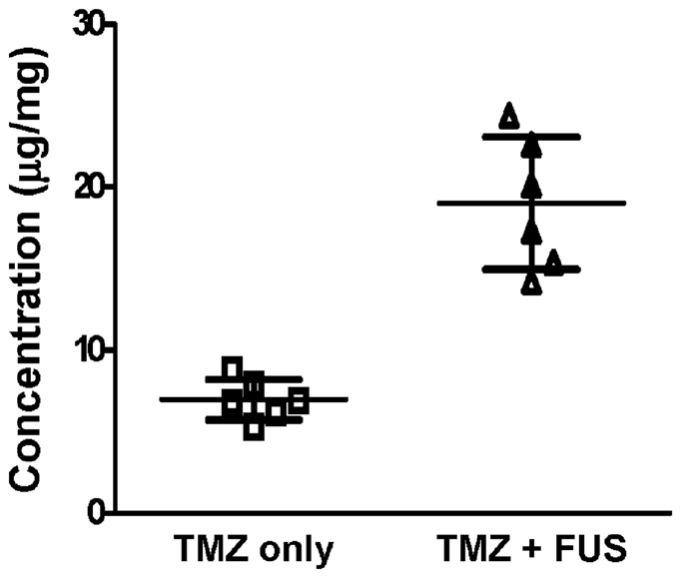
LC-MS/MS measurement of TMZ concentration (µg/mg) in FUS-BBB opened and contralateral brains (n = 6).

**Figure 4:**
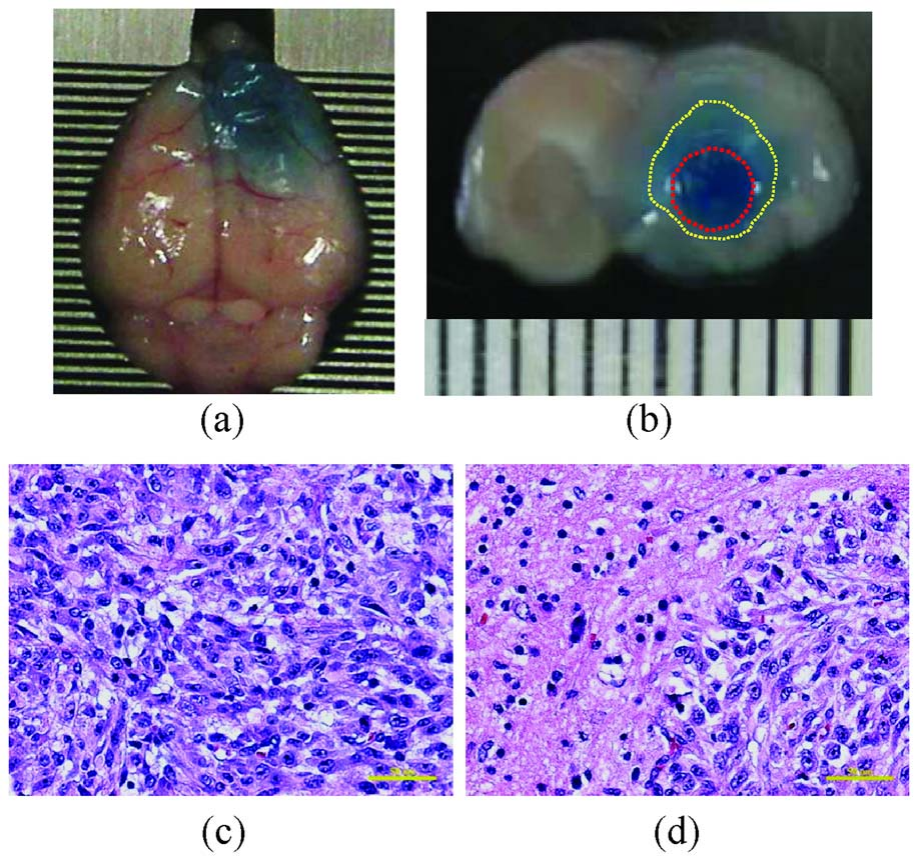
Representative Evans Blue (EB) dye staining in animal brains after FUS-BBB opening. (a) Top view; (b) cross-sectional view; (c, d) HE stains of FUS exposure and contralateral brains. White dashed contour  =  FUS-BBB-opened regions; Red dashed contour  =  tumor region. Bar  = 50 µm.

Next, we analyzed the dynamic TMZ concentration change due to FUS-BBB opening. The measured TMZ concentration in brain tissue of the FUS-BBB opening group was found to be 2.7-fold higher than that in the group that received TMZ alone (Fig. 3; 19±4.055 ng/mg versus 6.983±1.235 ng/mg, respectively). We analyzed the dynamic TMZ concentration changes in tumor-bearing mice to assess the pharmacodynamic change resulting from FUS exposure. Figs. 4(a) and 4(b) demonstrate typical examples of EB staining in tumor-bearing animals after FUS exposure. Dark EB staining (contoured in red) represents the tumor core region, whereas FUS-BBB opening created an outer rim of EB-stained regions with good coverage of the tumor (contoured in white). HE staining showed the morphology of the tumor core as well as the outer rims, revealing abnormal and leaky capillary structure accompanied by grouped tissue necrosis in the tumor core, but with normal pathological observation in the tumor rims (Fig. 4(c) and 4(d); comparison between substance leakage induced by tumor alone or enhanced by FUS exposure can be found in our previous study [22]).

**Figure 5:**
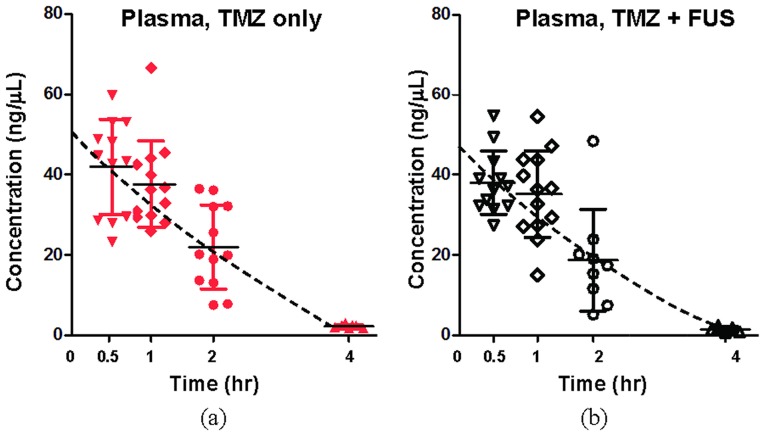
Concentration-time profile of TMZ measurements in plasma. (a) TMZ alone; (b) TMZ combined with FUS-BBB opening. A TMZ dose of 50 mg/kg was administered, and a FUS exposure power of 2 W was delivered in the TMZ + FUS animal group. Data are presented for individual measurements with the mean values for each group, and dashed line represents the corresponding progression curve estimation.

**Figure 6:**
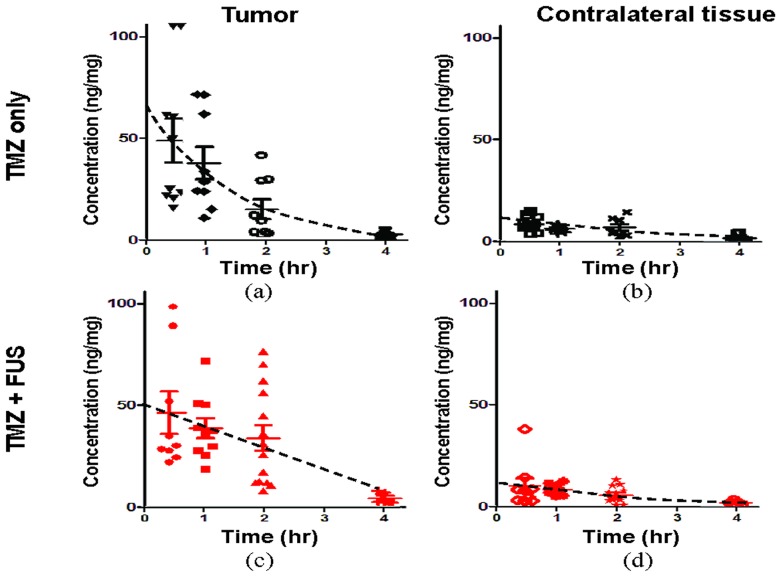
Concentration-time profile of TMZ measurements in brain tumor and contralateral brain tissue. (a, b) TMZ alone, tumor and contralateral tissue; (c, d) TMZ combined with FUS-BBB opening, tumor and contralateral tissue. A TMZ dose of 50 mg/kg was administered, and a FUS exposure power of 2 W was delivered in the TMZ + FUS animal groups. Data are presented for individual measurements with the mean values from each group, and dashed line represents the corresponding progression curve estimation.

Fig. 5 shows the TMZ concentration change over time measured from plasma for both TMZ-alone and TMZ+FUS groups, demonstrating that both TMZ concentration and pharmacodynamics in plasma were identical between the two groups (peak concentrations measured at 0.5 hrs were 41.933±11.831 and 38.014±7.874 ng/µL, respectively, and the concentrations degraded to 21.96±10.452 and 18.72±12.657 ng/µL, respectively, at 2 hours after administration), showing that FUS exposure did not alter TMZ dynamics in plasma. Similarly, TMZ concentration in contralateral brain was measured to be independent of FUS interventions (peak concentrations at 0.5 hrs were 8.953±3.453 and 10.733±11.1 ng/mg, respectively, and the concentrations degraded to 6.95±3.732 and 6.048±3.704 ng/mg, respectively, at 2 hours; TMZ concentration in the brain was 20–30% of the level in plasma) (Fig. 6).

**Figure 7:**
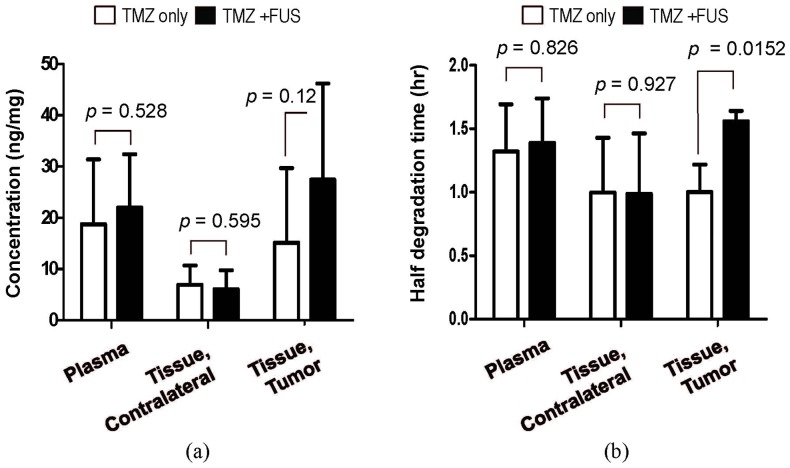
(a) TMZ concentration (mean ± STD) at 2 hours after TMZ administration obtained from plasma and brain tissues from each experimental group. (b) Estimated time (in hours) for TMZ to degrade to 50% of the peak level shown in Fig. 5.

On the other hand, for brain tumor regions, TMZ measurements were more diversified than those observed in the contralateral tissues and plasma. We found that FUS exposure induced an elevated TMZ level at 0.5 to 1 hr. Yet, the TMZ level was higher in the FUS exposure groups at 2 hours than the TMZ-alone groups (Fig. 6). Fig. 7(a) shows that the TMZ concentrations at 2 hours was about 2-fold higher in combined FUS exposure with TMZ administration when combined with TMZ administration alone, although the difference was not statistically significant (14.8 ng/mg versus 28.7429 ng/mg). We also analyzed the time it took for TMZ to degrade to 50% of its peak level and the results are summarized in Fig. 7(b). We found that the degradation times in plasma (1.32±0.37 and 1.39±0.35 hrs) and in contralateral brains (1.00±0.43 and 0.99±0.48 hrs) from TMZ alone and TMZ+FUS groups, respectively, were similar, and the measured values were comparable to those from previous studies [24]. TMZ in tumors degraded faster than in plasma and normal tissues (1.02±0.22 hrs). Of note, when combined with FUS-BBB, the degradation time was significantly prolonged to 1.56±0.08 hours (i.e., about a 1.5-fold increase).

In group 3 animals, we intended to evaluate the therapeutic effect when we combined FUS exposure with TMZ administration, but prior to this, we had to determine the TMZ dosing applied in glioma animals. S1 Figure shows the typical T2-MRI brain images obtained as well as the tumor volume/tumor growth ratio from each sub-group during a follow-up period (days 10–38). Generally, the tumor progressed with time after implant, and animals without TMZ administration showed the most aggressive tumor progression (control tumor volume was 158.26±104.85 and tumor progression ratio was 40.96±31.73 at day 38 after implantation). It was also noted that tumor progression control efficiency was strongly dependent on the dose of TMZ. A TMZ dose of 2.5 mg/kg only showed short-term progression suppression, whereas 5 mg/kg showed the most significant and long-term suppressive effect (S2 Figure). Animal survival was also highest with the high-dose TMZ group (median survival of 70 days) compared to the median- or low-dose TMZ groups (median survival of 40 and 52 days, respectively; S3 Figure).

**Figure 8:**
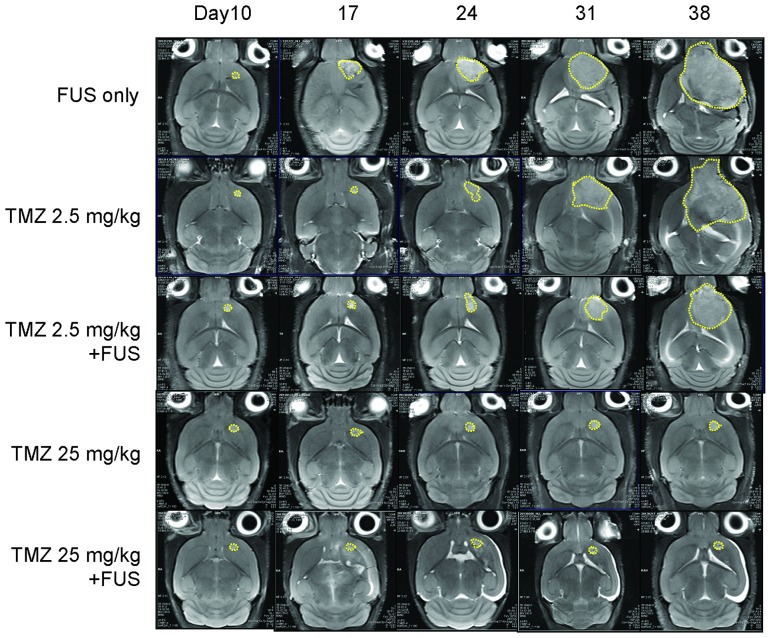
Representative T2-weighted MR images to monitor brain tumor progression weekly from days 10 to 38 in each of the subgroups of experimental group 3 (groups: sham control, FUS-BBB only, TMZ of 2.5 mg/kg (per day for 3 days) without and with FUS-BBB opening, TMZ of 25 mg/kg (per day for 3 days). Bar  = 0.5 mm.

**Figure 9:**
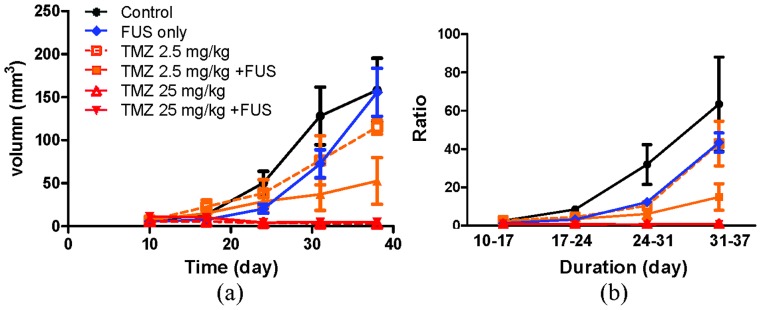
(a) Tumor progression (in volume; mm^3^) from days 10 to 38 in each subgroup from experimental group 3; (b) corresponding tumor progression ratio determined from (a) for a time period of 7 days.

We then investigated if the therapeutic efficacy of TMZ in the glioma animal model improved when TMZ administration was combined with FUS-BBB opening. Fig. 8 shows typical T2-MRI brain images obtained from each animal subgroup. The effect of FUS-BBB opening on tumor progression was analyzed and the results are shown in Fig. 9. While the use of FUS-BBB opening did not provide obvious long-term tumor suppression effects (tumor volume was 155.67±48.39 mm^3^ and the growth ratio was 43.41±11.33, which is similar volumes in the sham control group), the intermittent growth ratio at days 14–28 was found to be slower than that in the sham control group (p<0.05) and was almost equivalent to the 2.5 mg/kg TMZ suppression effect. The combination of FUS-BBB opening with 2.5 mg/kg TMZ administration effectively inhibited tumor growth (from 115.2±18.32 mm^3^ to 52.6±60.77 mm^3^ at day 38), and also resulted in a significant improvement in the tumor growth ratio reduction (from 42.87±26.17 to 14.95±15.51 at day 38 after implantation; p<0.05). On the other hand, 25 mg/kg TMZ alone and the combination of 25 mg/kg TMZ with FUS exposure provided nearly complete tumor progression suppression (tumor volume in both cases was nearly reduced to zero, and the tumor growth ratio was 0.98±1.02 and 0.76±0.56, respectively). Overall, FUS exposure provides extra therapeutic benefits over TMZ administration alone, especially for low TMZ dose.

**Figure 10:**
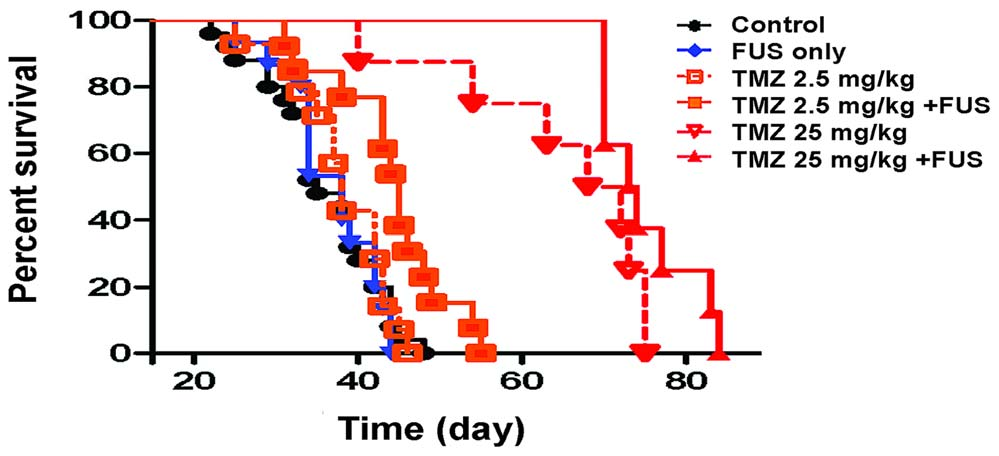
Kaplan–Meier plot demonstrating animal survival in experimental group 3.

We also analyzed animal survival and present the results in the Kaplan-Meier plot in Fig. 10. The median survival of the tumor-bearing animals without any interventions was 35 days. The animals that received FUS-BBB opening without administration of TMZ presented a similar survival trend (median survival  = 38 days), indicating that FUS BBB-opening alone did not post effective animal survival benefit. The administration of 2.5 mg/kg TMZ alone did not significantly improve animal survival (median survival  = 40 days; p = 0.1634), but the combination of FUS-BBB opening with 2.5 mg/kg TMZ significantly prolonged the animal survival (median survival  = 45 days). Compared to the survival in the control group, this was equivalent to a 14.3% IST_median_ when FUS-BBB opening was combined with TMZ administration. In contrast to 2.5 mg/kg, the 25 mg/kg TMZ delivery significantly improved the animal survival 2-fold (from 35 to 70 days in median survival; equivalent to IST_median_ of 14.3% to 28.6%, or IST_mean_ of 10.3% to 24.7%, when compared to the control animal group). In addition to the increased survival, combined FUS-BBB opening also produced a further gain in median survival of up to 73.5 days (equivalent to IST_median_ of 100% to 111.4%, or IST_mean_ of 77.7% to 108.6%, when compared to control animal group). Of note, the FUS-BBB opening with 25 mg/kg TMZ administration increased survival in all animals to longer than 70 days, whereas the same occurrence was found to be only 50% in the animals that received 25 mg/kg TMZ only. Animal survival analysis was summarized in Table 1. All raw analytical data presented in this study were listed in S1 Data.

**Table 1:** Efficacy of various treatment protocols for induced brain tumors in mice.

Group (n)	Median survival (days)	IST_median_ (%)	Mean survival (days)	IST_mean_ (%)	*p-value*
**Control (27)**	35	—	36.0±6.9	—	—
**FUS only (16)**	38	8.6	37.3±5.8	3.6	0.8255
**TMZ, 2.5 mg/kg (18)**	40	14.3	39.7±6.6	10.3	0.1634
**TMZ, 2.5 mg/kg +FUS (17)**	45	28.6	44.9±7.5	24.7	<0.05
**TMZ, 5 mg/kg (13)**	52	48.6	53.6±7.6	48.9	<0.05
**TMZ, 25 mg/kg (8)**	70	100	64.0±11.9	77.7	<0.05
**TMZ, 25 mg/kg +FUS (8)**	74	111.4	75.1±5.7	108.6	<0.05

Increase in median survival time (IST_median_; in %), increase in mean survival time (IST_mean_; in %), *p*-values are all relative to the control group.

## Discussion

In this study, we performed pharmacodynamic analysis of combined TMZ administration with FUS-induced BBB opening to improve glioma treatment. This is the first study to report that FUS-BBB opening can effectively increase TMZ concentration and alter pharmacodynamics in local targeted brain tumor tissues (2.75-fold concentration increase), thereby prolonging the presence of TMZ (1.5-fold increase in TMZ degradation time) in tumor regions. This is particular of interested in GBM patient treatment since peripheral glioma has been shown to remain highly functional with an intact BBB and usually plays a critical role in tumor recurrence [13], [14], [27]. The intact BBB of tumor-infiltrating regions (mostly at the tumor periphery) severely restricts treatment efficacy, contributing to the high rate of GBM recurrence. For this reason, enhancing the BBB permeability of the tumor periphery represents an important strategy for improving treatment efficacy. FUS-BBB opening creates an opportunity to increase TMZ concentration in the tumor periphery, which might provide better tumor progression and recurrence control.

Brain tumors are usually most permeable in the tumor core, whereas the BBB remains relatively intact in the tumor periphery [12]. Similarly in this study, we observed that the peak TMZ concentration did not increase at the implant tumor core (41.18±31.1 vs. 48.93±34.14 ng/mg). Nevertheless, the TMZ degradation time in the tumor core was significantly delayed from 0.67±0.22 to 1.56±0.08 hours (i.e., 2.32-fold increase), and TMZ concentration still remained high when compared to the concentration in the control tumor core. We postulate that the delayed TMZ degradation effectively increases the area under the curve (AUC), thereby contributing to a total TMZ accumulation and interaction in the tumor core and offer gained therapeutic efficacy of TMZ to against brain tumors.

Quantification and pharmacokinetic analysis of TMZ is always challenging in biological tissues due to the instability and fast degradation of TMZ. In aqueous buffers, TMZ is stable at pH<4, but it rapidly decomposes to MTIC at pH>7. MTIC, on the other hand, is stable at alkaline pH, but rapidly breaks down to AIC at pH<7 [5], [6], [28], [29]. The *in vitro* half-life of TMZ in phosphate buffer at pH 7.4 is 1.9 hours at 37°C compared to only approximately 2 min for MTIC at the same temperature, and up to about one hour for MTIC when the temperature is decreased to 4°C [24]. Our previous reports confirmed that FUS-induced BBB opening could enhance the CSF/plasma ratio of TMZ by 70% in an animal model (from 22.7% to 38.6%), but the direct measurement of TMZ in brain tissues was not feasible at the time of prior studies. The TMZ concentration after diffusion into the CSF from tissue is likely an underestimation of the actual accumulation of TMZ in brain tumor, so it has been postulated that TMZ concentration improvement in brain tissue could be more significant. In this study, we used LC-MS/MS, where the pH could be precisely controlled immediately after sample collection and preparation, to successfully quantitate how FUS-BBB opening enhances TMZ concentration in tissue and plasma, and confirmed the TMZ concentration increase.

Also, following oral administration, a previous report revealed that TMZ reached its peak concentration at about 0.33 to 1 hours, with an estimated half-life of about 1.8 hours [30]. TMZ is most commonly quantitated by high-performance liquid chromatography (HPLC) [4], [5], [24], MS/MS analysis [31] or radiolabel/radioactivity detection [32] in plasma. Our proposed LC/MS approach primarily estimated that degradation of TMZ to 50% its concentration took 1.4 hours in plasma, which is close and comparable with the previously reported half-life, and supports the accuracy of this TMZ quantification approach. With the FUS exposure, we observed that the mean tumor concentration increased from 15.13±14.57 to 27.43±18.77 ng/mg at 2 hrs. The elevated TMZ concentration was considered to be the explanation for the extension of the estimated 50% TMZ degradation time from 1.02±0.22 to 1.56±0.08 hours (i.e., 1.53-fold increase), suggesting that the half-life of TMZ in brain tumor can be regulated and prolonged when FUS exposure is involved.

Enhanced therapeutic efficacy in TMZ delivery when combined with FUS-BBB opening is supported by our observation of tumor progression control (maximal tumor progression ratio of 42.87 and 0.98% in low and high TMZ dose administration), and increased survival (maximal survival improvement up to 14.3% and 100% when administering low and high TMZ dose). The FUS-induced pharmacodynamic changes and the corresponding half-life characterization should be investigated in more detail in future studies.

Although the clinically suggested TMZ administration in GBM patient treatment is 200 mg/m^2^/day with a consecutive 5-day delivery [7], [8], previous mice glioma models have shown that a dose of 65.78 mg/m^2^/day over a five-day period can result in efficacious treatment [33]. Also, the TMZ dose ranging from 5 to 75 mg/m^2^/day has been selected to evaluate the synergistic tumor suppression from combined modalities for TMZ delivery to human glioma cell implants into nude mice [34], [35]. In this study, we mimicked the reported TMZ dose range to test the synergistic treatment efficacy of FUS-BBB opening for enhanced local TMZ deposition into the tumor (the applied 2.5 to 25 mg/kg/day is equivalent to 8 to 80 mg/m^2^/day; transferred by using body surface area (BSA) of 80 cm^2^ in 25 g mice). In this study, we confirmed that FUS-BBB opening can increase chemotherapeutic efficacy with a wide range of TMZ levels, thereby supporting the use of FUS exposure to enhance TMZ delivery at different tumor stages.

## Conclusion

Here we show that transcranial FUS-BBB opening treatment enhances the delivery of TMZ through the BBB, enabling targeted increase in chemotherapeutic drug dosage and induce targeted pharmacodynamic change of TMZ in the tumor region. FUS-enhanced delivery of TMZ significantly suppressed tumor growth and prolonged animal survival, suggesting that this approach may improve future therapeutic outcomes from TMZ chemotherapy for brain tumors. Because TMZ is the first-line chemotherapeutic drug for treatment of GBM, this procedure could be highly clinically relevant, with the potential to ultimately advance the use of chemotherapy to treat patients with central nervous system malignancies. Our findings encourage further in-depth exploration of the benefits of locally increasing the concentrations of chemotherapeutic drugs for the most effective treatment of brain tumors.

## Supporting Information

S1 Figure **Representative T2-weighted MR images to monitor brain tumor progression weekly from days 10 to 38 in each subgroup of experimental group 2.** (a) Sham control; (b) TMZ of 2.5 mg/kg (per day for 3 days); (c) TMZ of 5 mg/kg (per day for 3 days); (d) TMZ of 25 mg/kg (per day for 3 days). Bar  = 0.5 mm.
(TIF)

S2 Figure **(a) Tumor progression (in volume; mm^3^) from day 10 to day 38 for each sub-groups in experimental group 2; (b) Corresponding tumor progression ratio determined from (a) for a time period of 7 days.**
(TIF)

S3 Figure **Kaplan–Meier plot demonstrating animal survival in experimental group 2.**
(TIF)

S1 Checklist **ARRIVE Checklist.**
(PDF)

S1 Data **Raw analytical data presented in this study.**
(XLS)
