# Supplementary figures and images for: Pharmacodynamic and Therapeutic Investigation of Focused Ultrasound-Induced Blood-Brain Barrier Opening for Enhanced Temozolomide Delivery in Glioma Treatment
Source: PLoS One. 2014 Dec 9;9(12):e114311. doi: 10.1371/journal.pone.0114311 (PMC4260869; doi:10.1371/journal.pone.0114311)

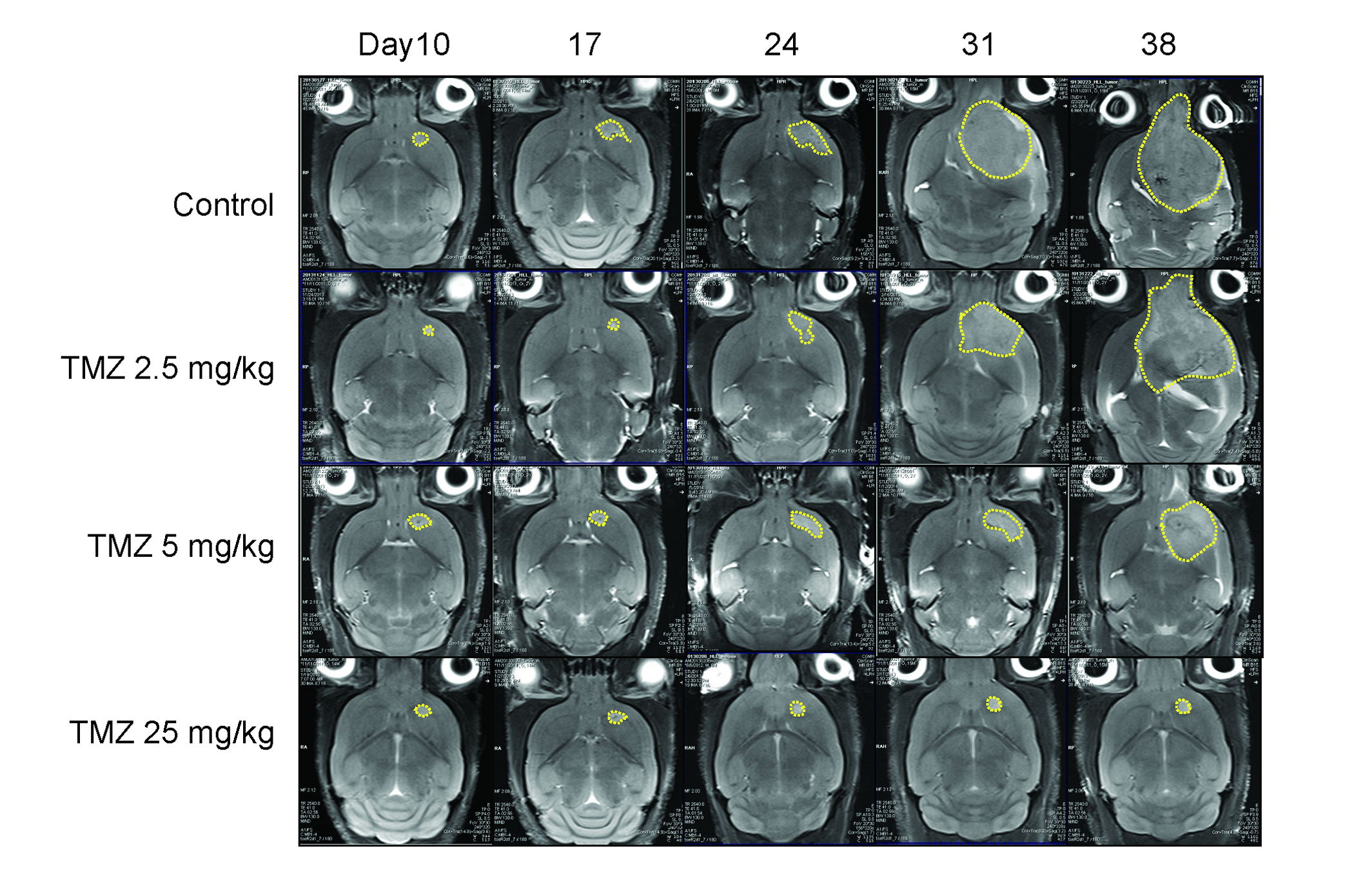

Supplement: S1 Figure — Representative T2-weighted MR images to monitor brain tumor progression weekly from days 10 to 38 in each subgroup of experimental group 2. (a) Sham control; (b) TMZ of 2.5 mg/kg (per day for 3 days); (c) TMZ of 5 mg/kg (per day for 3 days); (d) TMZ of 25 mg/kg (per day for 3 days). Bar = 0.5 mm. (TIF) [file pone.0114311.s001.tif]

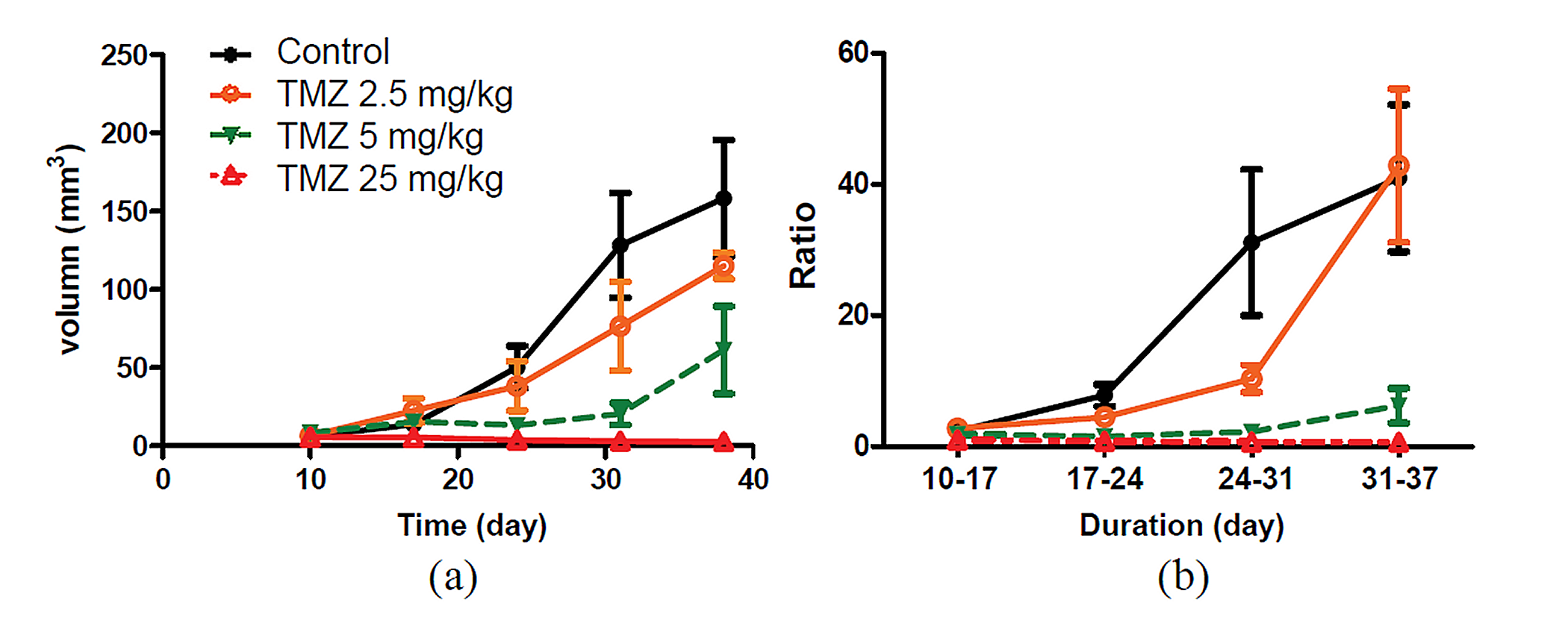

Supplement: S2 Figure — (a) Tumor progression (in volume; mm3) from day 10 to day 38 for each sub-groups in experimental group 2; (b) Corresponding tumor progression ratio determined from (a) for a time period of 7 days. (TIF) [file pone.0114311.s002.tif]

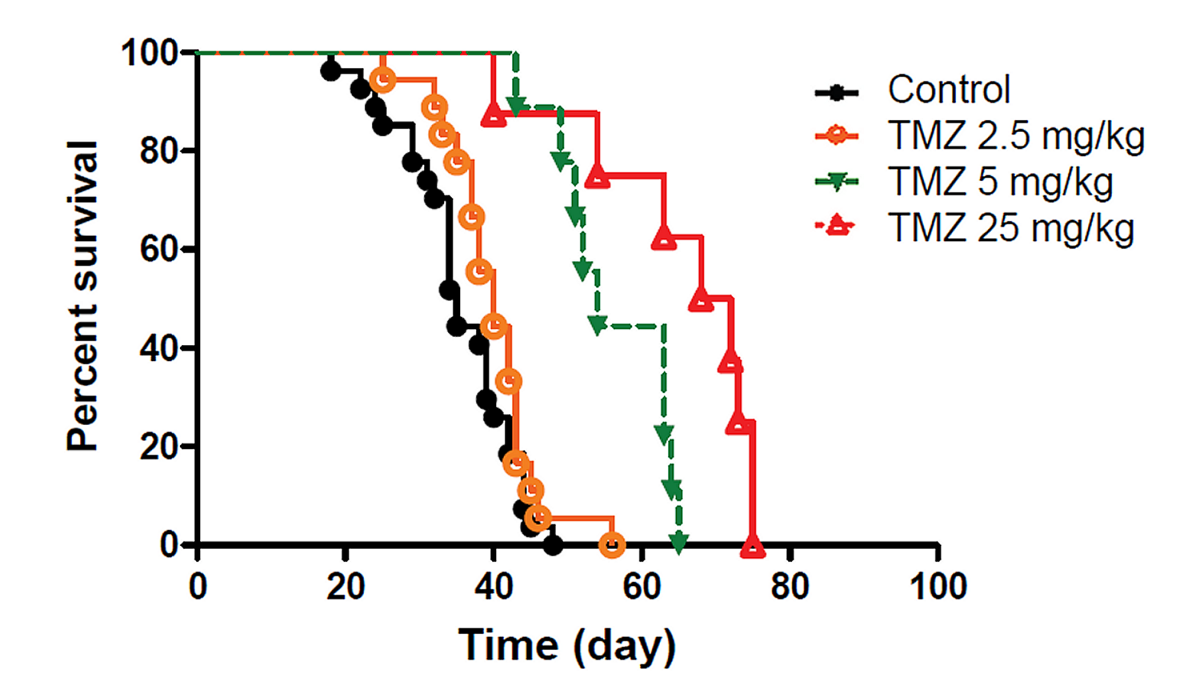

Supplement: S3 Figure — Kaplan–Meier plot demonstrating animal survival in experimental group 2. (TIF) [file pone.0114311.s003.tif]
